# Effects of short-term existential group therapy for breast Cancer patients

**DOI:** 10.1186/s13030-021-00225-y

**Published:** 2021-11-27

**Authors:** Chizu Nakamura, Masatoshi Kawase

**Affiliations:** 1 Department of Psychology, Kyoto Notre Dame University, 1 Minami Nonogami-cho, Shimogamo Sakyo-ku, Kyoto, 606-0847 Japan; 2 Department of Psychology, Osaka University of Human Sciences, 1-4-1 Shoujyaku. Settu, Osaka, 566-8501 Japan; 3 Department of Psychiatry, Daigo Hospital, 72 Ishida Oyamachou, Fushimi-Ku Kyoto, 601-1433 Japan

**Keywords:** Group therapy, Spiritual well-being, Existential, Hopelessness, Breast cancer

## Abstract

**Objectives:**

Cancer patients who suffer from existential difficulties, including fear of death, isolation, or loss of human relationships, try to accept these fears by exploring the meaning of their life. In particular, early psychological intervention for patients prevents them from psychosocial maladjustment afterwards. Therefore, we have developed the Short-term Existential Group Therapy Program (Short-term EGP) for cancer patients, focusing on relief of existential or spiritual suffering and/or pain. This study aims to statistically evaluate the effects of this program on breast cancer patients within the first year after cancer diagnosis.

**Methods:**

Thirty-one patients completed our research program. A ninety-minute therapeutic group session was held once a week for 5 weeks. We performed the above assessments three times: just before and after the intervention, as well as a month after the end of intervention. Outcome assessment included measures of spiritual well-being (SELT-M), Mental Adjustment to Cancer (MAC) and Profile of Mood States (POMS).

**Results:**

The SELT-M “Overall QOL” scores were significantly increased after intervention, and these scores were maintained a month after intervention, particularly in those with high MAC “Hopelessness” scores. Subscales of the SELT-M scores were significantly increased after intervention, and these scores were maintained up to a month after intervention.

**Conclusion:**

Short-term EGP intervention could be effective in helping patients relieve their existential distress. Some of the treatment effects were maintained a month after the end of the intervention. In addition, Short-term EGP could be particularly effective for those patients who feel hopelessness after cancer diagnosis.

**Trial registration:**

Retrospectively registered. University Hospital Medical Information Network (UMIN CTR) UMIN000040651. Registered June 4, 2020.

## Introduction

When diagnosed with cancer, 30-50% of cancer patients experience psychiatric symptoms, such as anxiety and depression [1–3]. Psychiatric symptoms are associated with physical, psychological, social, and existential pain, which affect patient quality of life (QOL) [4]. These psychiatric symptoms not only affect the QOL, but they can also be a factor in suicide [5]. In particular, the risk of suicide in cancer patients within 1 year after diagnosis is high [6, 7]. The hopelessness resulting from existential pain, including fear of death, isolation, or loss of human relationships, is a predictive factor of suicidal ideation in cancer patients [8]. Therefore, early detection and intervention of psychiatric symptoms such as anxiety and depression related to existential distress in cancer patients is clinically important.

Psychotherapy via individual and group therapy intervention focusing on existential pain was reported to relieve anxiety and depression and to improve spiritual well-being in cancer patients in the West [9–12]. Furthermore, it was demonstrated to be effective for improving the QOL of cancer patients in the terminal and early stages [13–17]. There are few individual interventions or group therapies that address existential pain in cancer patients at stages (acute or chronic) other than terminal in Japan [18–25]. Our previous study revealed that Japanese cancer patients who are relatively independent except in the terminal stage have existential issues [26].

Group therapy is effective at easing the feeling of isolation of patients. Participants for group therapy can be models for others, exchange information, and learn how to cope with cancer by socially supporting each other [27]. In addition, group therapy may benefit both patients and clinicians in labor, time, and medical expenses. Because of these advantages, we used group therapy in this study. Kissane [14] developed Cognitive-Existential Group Psychotherapy, a 20-week intervention for women with breast cancer. This therapy had 6 goals: promoting supportive environment, facilitating grief over losses, cognitive reframing, problem solving, fostering hope, and examining priorities for the future. They reported that this intervention tended to reduce anxiety and that the participants had significantly greater satisfaction with their therapy. However, some patients may find it burdensome to participate in the treatment every week for 5 months. Therefore, in order to make it easier for more patients to participate, we believe it is better to shorten the number and duration of treatments. Label [16] developed a 6-week cognitive-existential group intervention for women with breast cancer or ovarian cancer that focused on fear of cancer recurrence. This intervention focused on patients who had high -fear of cancer recurrence and who completed their first-line treatment (i.e., surgery, radiation, or chemotherapy). Breitbart [9] developed Meaning Centered Group Psychotherapy, an 8-week intervention for patients with advanced cancer. This therapy focused on enhancing a sense of meaning, peace, and purpose in their lives to improve spiritual well-being. These two therapeutic interventions focus on existential issues and have been shown to improve anxiety and spiritual well-being in cancer patients. However, the target populations of the interventions are those with high recurrence anxiety and advanced cancer patients. On the other hand, we have shown that cancer patients in the early stage after diagnosis also have existential problems [26].

We believe that even if there are no clinical signs of psychological morbidity, such as depression and anxiety, early relief of existential distress is important to prevent it from evolving into depression and anxiety, which require clinical intervention, so we hoped to develop a group therapy that can be adapted to cancer patients with an early stage after cancer diagnosis. To this end, we have developed a short-term group therapy program for cancer patients that focuses on relief of existential or spiritual suffering and/or pain. This study evaluated the effects this program for patients with cancer at stages other than terminal. We focused on breast cancer patients because homogenization is important to examine the effects of a new program.

## Methods

### Participants and procedures

Subjects were recruited from two clinics that specialize in breast cancer in Shiga and Kyoto Prefectures, Japan.

Criteria for inclusion were: having been diagnosed with breast cancer within twelve months prior to enrolling in the study, having had breast surgery (conservative or mastectomy), diagnosis of breast cancer at disease onset, 30-65 years old, and note of whether they had received adjuvant chemotherapy. Because this program did not focus on issues specific to the AYA generation, the target population for this study was defined as 30 years of age or older. In addition, subjects were screened with the Hospital Anxiety and Depression Scale (HADS) [28]; those scoring under ten points on the depression scale (from 0 to 21) were included. All participants provided informed consent, and then we performed this program after asking patients to answer the questionnaire. This study was approved by the ethics committee of the Department of Psychology of Kyoto Notre Dame University. This study was conducted from 2008 to 2013.

### Short-term existential group therapy program (short-term EGP)

We developed what we call the Short-term Existential Group Therapy Program (Short-term EGP) for cancer patients [29], a 90 min therapeutic group session that is held once a week for 5 weeks. Each session consists of between five to seven participants with a psychiatrist and clinical psychologist as facilitators and with a specific subject of discussion, as described below (1st), 2nd), 3rd), 4th), 5th)).

The participants were encouraged to talk freely about themselves and any existential anxieties they may have, such as a sense of helplessness or fear of losing a peaceful life, which were shared among the participants. The patients could benefit from the presence of professionals whose basic suggestions were based on the encouragement of the patients to find social support from others. While the patients spoke and listened to others during the sessions, they were prompted to self-reflect and encouraged to believe in themselves.

1st) Talk freely about yourself.

2nd) Share your anxieties. Be conscious of the changes the illness may have on your mind and body, and be aware of your emotions and condition. Express anxieties that stem from confrontation and uncertainty of your illness, and share them.

3rd) Re-construct human relationships. Consider how to communicate with your family, friends, and other people who you are in conflict with or you feel ambivalent about.

4th) Cope with the stresses. Talk concretely about how to face stresses so that you may cope with anxieties and things you may be worried about.

5th) Know who you are. Consider what is important, be aware things that are intimate consistently to you in terms of the past, present, and future, by finding meaning and purpose in your life.

### Measures

#### Socio-demographic and cancer-related information

The patients provided their age, gender, marital status, and family constitution by self-report. We also requested information regarding the stage of cancer, treatment, and metastasis of each subject.

#### Profile of mood states - brief form (POMS)

The POMS [30] was used to evaluate patient mood state. It comprises 30 items and 6 subscales**:** “Tension-Anxiety”, “Anger-Hostility”, “Confusion”, “Fatigue”, “Depression”, and “Vigor”.

Each item is scored from 0 to 4 points, with a lower score indicating poorer health.

#### Skalen zur Erfassung von Lebensqualität bei Tumorkranken (SELT-M)

The SELT-M [31] was used to evaluate spiritual well-being. It is a 15-item questionnaire employing a 4-point scale (1-4), with higher scores indicating greater spiritual well-being. A single-item sub-scale “Overall QOL” asked for self-evaluation of the participant’s present spiritual state by a numerical scale from ten (highest) to zero (lowest). Other subscales included were “Orientation (3 items)”, “Spirituality (8 items)”, and “Support (3 items)”.

#### Mental adjustment to Cancer (MAC)

The MAC [32] was used to evaluate patient coping with cancer.

The MAC employs a 4-point scale (1-4) and consists of 5 subscales: “Fighting spirit”, “Anxious preoccupation”, “Hopelessness”, “Fatalism”, and “Avoidance”.

#### Description of semi-structuring

Questionnaire consisting of statements to be completed by describing the meaning and purpose of life based on one’s experience:

The questionnaire consists of 8 statements that refer to Parts A and B of the Japanese-version Purpose in Life Test [33]: {Daily life (work, housekeeping) to me seems:}, {Life to me seems:}, {I am a:}, {In thinking of my life, I:}, {Every day is:}, {Illness and distress are:}, {If I could choose, I would:}, and {My life goals are:}. The latter part of each statement is described by respondents to complete the statement.

#### Term of evaluation

We performed the above assessments three times: just pre and post the intervention (after 5 session) and a month after the end of intervention.

### Data analysis

#### Quantitative analysis

Statistical analyses were performed using the IBM SPSS 22.0 J package for Windows. Effects of therapy were assessed under an open trial design.

For spiritual well-being and emotional distress, mixed two-way repeated measures ANOVA was used, with coping styles for cancer (MAC subscales, divided into low or high-scoring subjects) as the between-subject factors, and time as the within-subject factor. Post hoc tests were used to compare scores obtained at the three time points, comparing pre-intervention and post-intervention scores, as well as post-intervention and 1-month assessment scores.

#### Qualitative analysis

Factors relieving existential distress and increasing the QOL were examined using analyzable descriptions of the meaning and purpose of life based on the experiences of 25 respondents. The questionnaire consisted of 8 statements to clarify their perceptions of life, illness, and themselves and attitudes toward life purposes. Descriptions for each questionnaire subscale at 3 points: before (P1), after (P2), and 1 month after (P3) intervention, were classified based on their content and categorized by 2 researchers, adopting the KJ method.

## Results

Thirty-four patients were enrolled for study, 31 of whom completed the intervention (91.2%). Their mean age was 50.5 years (range 32 to 65 years). All were women, 81% were married, and 19% were unmarried or divorced. Twenty-three patients underwent breast-conserving surgery and the other eight underwent mastectomy, 61% had no metastasis, 26% showed spread to lymph nodes, and 6% showed spread to bone.

The mean and standard deviation (SD) of the subscale scores of the Mental Adjustment to Cancer (MAC) assessment, divided into low- and high-scoring groups (LG,HG) were 52.53 ± 4.40 (HG) and 39.14 ± 4.67(LG) for “Fighting spirit (score range:16-64)”, 12.77 ± 1.78(HG) and 7.35 ± 1.22(LG) for “Hopelessness (score range:6-24)” , 28.07 ± 2.89 (HG) and 20.00 ± 2.26(LG) for “Anxious preoccupation (score range:9-36)”, 23.08 ± 3.30 (HG) and 14.38 ± 2.45(LG) for “Fatalism (score range:8-32)” , and 3.63 ± .52(HG) and 1.48 ± .51(LG) for “Avoidance (score range:1-4)” .

Correlation coefficients among the assessment subscales are shown in Table 1.

**Table 1:**
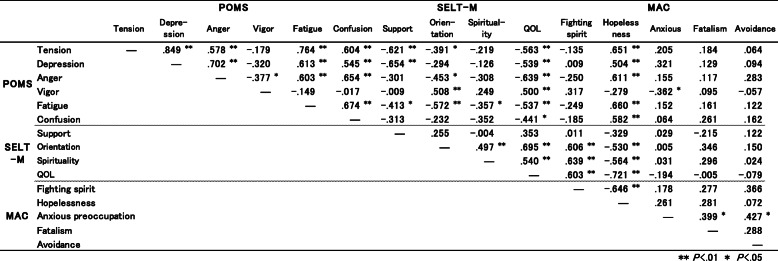
Correlations among psychosocial mesures

MAC-Hopelessness was significantly associated with Tension (*r* = .65; *p* = 0.000), Depression (*r* = .50; *p* = 0.005), Anger (*r* = .61; *p* = 0.000), Fatigue (*r* = .66; *p* = 0.000), and Confusion (*r* = .58; *p* = 0.001) of the POMS assessment, and with Overall QOL (*r* = .72; *p* = 0.000), Orientation (*r* = .-53; *p* = 0.003), and Spirituality (*r* = .-56; *p* = 0.001) of the SELT-M assessment. MAC-fighting spirit was significantly associated with Overall QOL (*r* = .60; *p* = 0.001), Orientation (*r* = .61; *p* = 0.000), and Spirituality (*r* = .64; *p* = 0.000) of the SELT-M assessment.

### Impact of intervention on spiritual well-being

To analyze the impact of intervention on spiritual well-being (SELT-M subscale), a mixed, two-factor repeated measures ANOVA was performed, with coping style for cancer (MAC subscales divided into low- and high-scoring groups) as the between-subject factors, and time as the within-subject factor (Table 2).

**Table 2:**
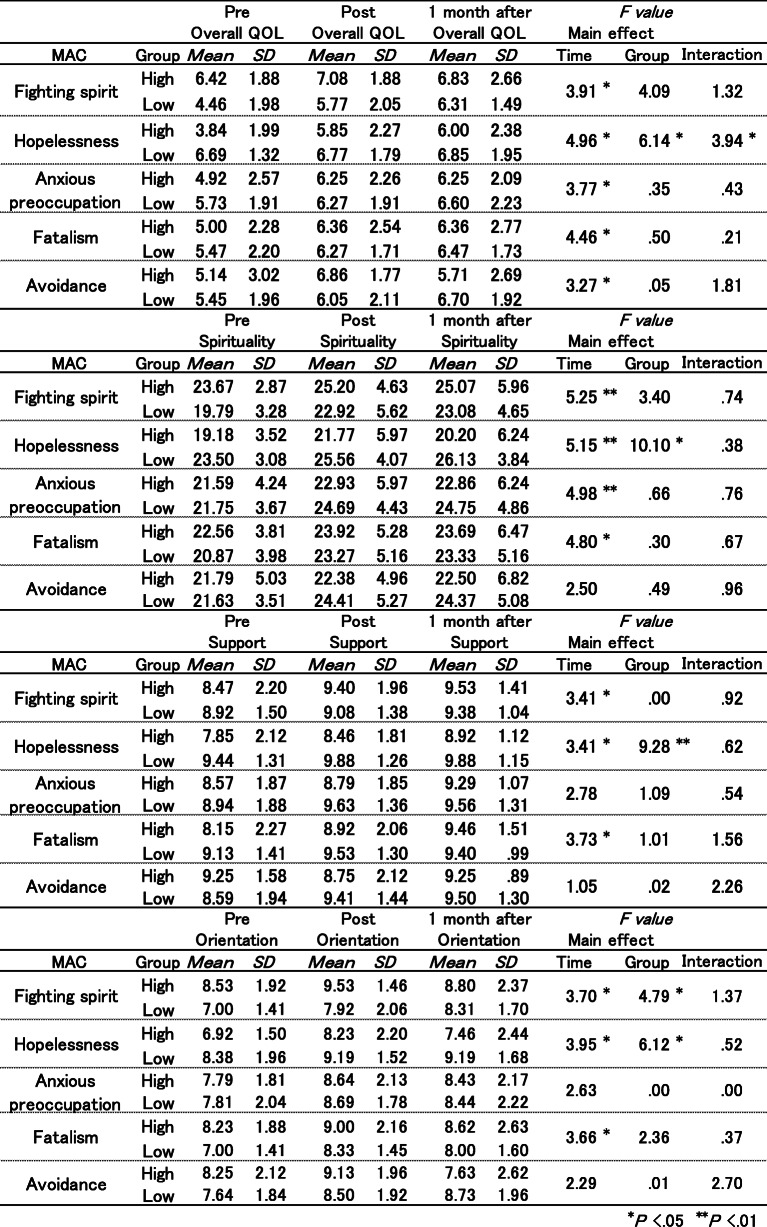
Two way ANOVA with MAC×SELT-M

**Fig. 1:**
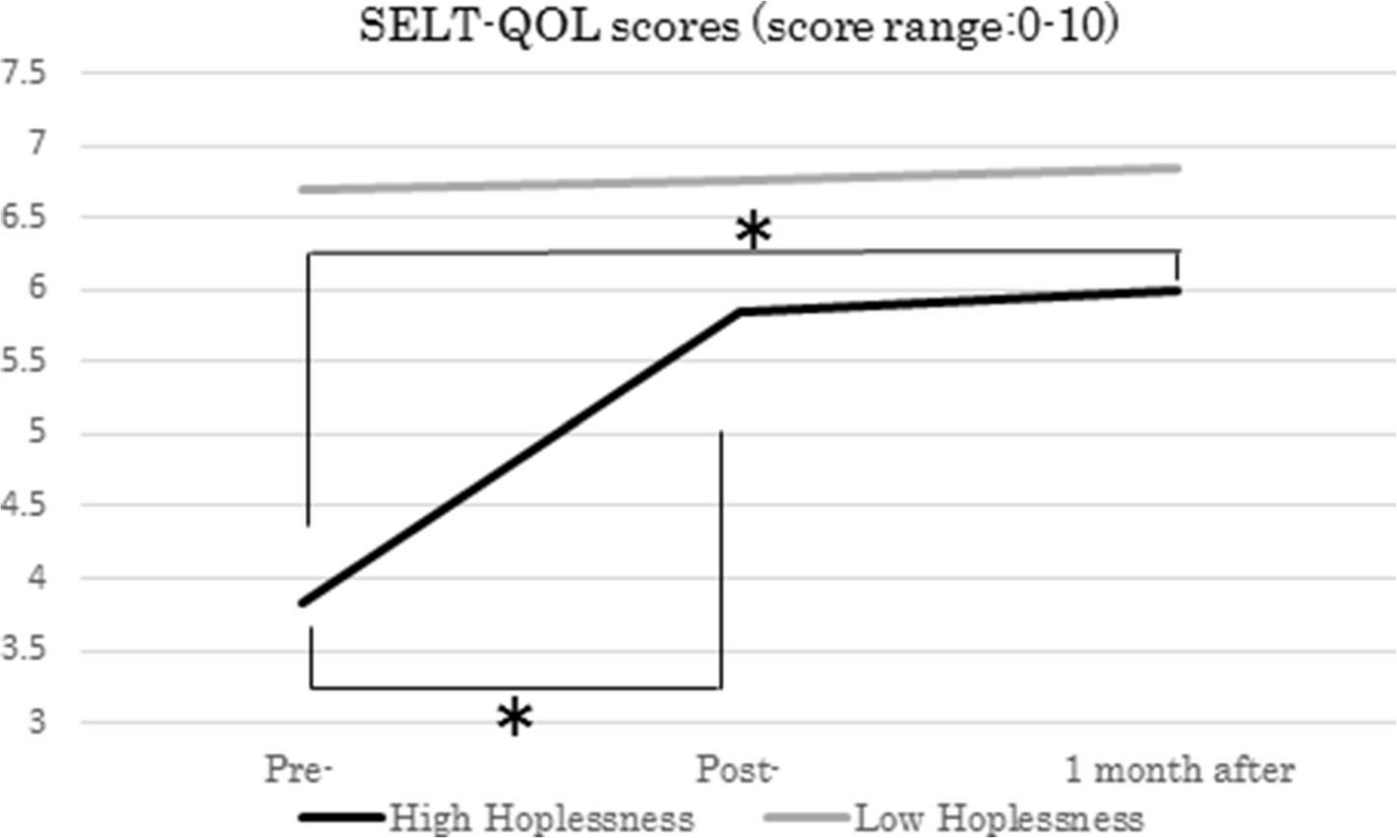
MAC “Hopelessness” and SELT-M “QOL”

For SELT-M “Overall QOL”, significant interaction was observed with the MAC “Hopelessness” subscale (*F* (2, 48) =3.94, *p* ≺ .05). As a result of the simple main effect, SELT-M “Overall QOL” scores were significantly increased after intervention, and these scores were maintained a month after intervention, particularly in those with high MAC “Hopelessness” scores (*F* (1, 24) =6.14, *p* ≺ .05 (Fig. 1)).

For SELT-M “Spirituality”, no significant interactions were identified among any of the factors. However, time was identified as a significant main effect on MAC subscale scores. The SELT-M “Spirituality” scores were significantly increased after intervention, and these scores were maintained up to a month after intervention. A similar pattern of findings was observed in the analysis of the “Support” and “Orientation” subscales of the SELT-M. No significant interactions were observed in either of the factors. However, except for the subscale “Avoidance”, time was identified as a significant main factor of the MAC subscale scores.

### Impact of intervention on emotional distress

The means and SDs of the POMS subscale scores (score range:0-20) were as follows: the average intensity of “Tension-anxiety” was 7.83(*SD* = 4.59); the average intensity of “Depression” was 5.00(*SD* = 4.40); the average intensity of “Anger-Hostility” was 3.66(*SD* = 2.66); the average intensity of “Vigor” was 6.03(*SD* = 3.32); the average intensity of “Fatigue” was 6.86(*SD* = 5.01); and the average intensity of “Confusion” was 5.52(*SD* = 2.81).

To analyze the impact of intervention on emotional distress (POMS), a mixed, two-factor repeated measures ANOVA was performed, with coping style for cancer (MAC subscales divided into low- and high-scoring subjects) as the between-subject factors, and time as the within-subject factor.

No statistically significant effects due to intervention were observed on the POMS subscale scores.

### Qualitative analysis

To identify factors relieving existential distress and increasing the QOL, descriptions of [the meaning and purpose of life] based on experiences of the respondents were analyzed. The associations among <category>, <Hopelessness> (2 MAC-based groups: High- and Low-hopelessness), and < QOL> (2 SELT-M-based groups: High- and Low-QOL) were examined by performing multiple correspondence analysis for each questionnaire subscale. In correspondence analysis, the closer the distance between categories, the higher the similarity between the items. Through the analysis for the High-hopelessness group, two statements {Daily life to me seems:} and {The purpose of my life is:} yielded notable results.

**Fig. 2:**
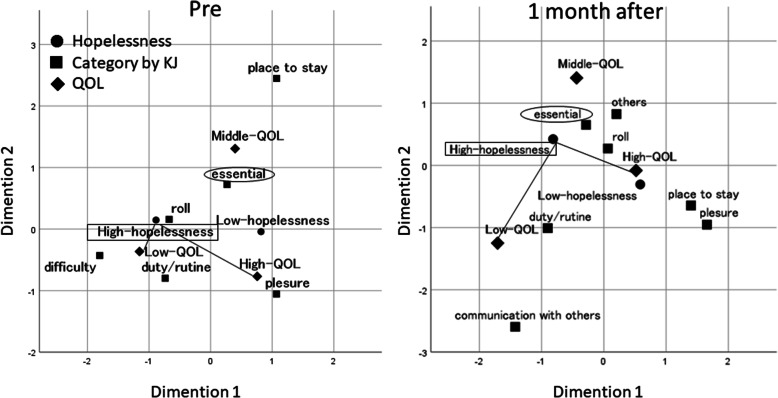
Multiple correspondence analysis correspondence map

As shown in Fig. 2, the following variables were identified at P1 and P3 through classification of descriptions for {Daily life to me seems:} adopting the KJ method: P1: <duty/routine>, <place to stay>, <own benefit>, <role>, <essential>, <difficulty>, and < pleasure>; and P3: <duty/routine>, <place to stay>, <role>, <essential>, <pleasure>, <communication with others>, and < others>.

The distances between the High-hopelessness (P1: x = − 0.887, y = 0.105; and P3: x = − 0.815, y = 0.422) and High-QOL (x = 0.756, y = − 0.860; and x = 0.517, y = − 0.082) groups, and between the High-hopelessness (x = − 0.887, y = 0.105; and x = − 0.815, y = 0.422) and Low-QOL (x = − 1.154, y = − 0.322; and x = − 1.707, y = − 1.251) groups at P1 and P3 were 1.88: 0.58 and 1.42: 1.90, respectively. Thus, the distance between the High-hopelessness and High- QOL groups was shorter at 1 month after intervention, representing their closer association.

Related factors were examined, focusing on <essential> and < duty/routine>. At P1, <duty/routine> (x = − 0.737, y = − 0.616, distance = 0.74) was closer to the High-hopelessness group than <essential> (x = 0.269, y = 0.550, distance = 1.24). In contrast, at P3, <essential> (x = − 0.285, y = − 0.650, distance = 0.58) was more closely associated with the group than <duty/routine> (x = − 0.902, y = − 1.01, distance = 1.43). <essential> also became closer to the High-QOL group at P3 (distance at P1 and P3: 1.49 and 1.23, respectively), suggesting that <essential> increased the QOL in the High-hopelessness group. <difficulty>, which was associated with the High-hopelessness group at P1, did not appear after intervention.

Furthermore, by similarly analyzing descriptions for {My life goals are:}, <smile> was also found to increase the QOL in the High-hopelessness group.

## Discussion

Cancer patients are at risk of anxiety and depression within the first year of cancer diagnosis [34, 35]. These psychiatric symptoms will continue to affect the patient’s QOL in the fight against cancer [36]. Furthermore, the risk of suicide in cancer patients within 1 year after diagnosis is high [6, 7]. Relieving existential distress at an early stage after onset can improve psychological morbidity such as depression and anxiety.

Previous studies demonstrated that hopelessness can negatively affect psychological well-being [37, 38] and cancer prognosis [39, 40] and lead to desire for hastened death (DHD) [8, 41, 42]. Our study showed hopelessness, which constituted existential distress, was significantly associated with emotional distress, including depression and spiritual well-being (Table 1). Patients identified with hopelessness significantly increased in SELT-M “Overall QOL”scores after intervention, which were maintained a month after the end of intervention (Fig. 2). Thus, our results suggest that short-term EGP could be effective in relieving existential distress in patients, especially for those feeling hopeless after cancer diagnosis. That is, group therapy program focusing on existential issues can be expected to be effective not only in the West but also in other areas of the world.

We attempted to identify factors relieving existential distress and increasing the QOL. The program led to cognitive changes in daily life and life purposes. Such changes may help to improve the QOL. These improvements may help stabilize the emotional state of a patient as well avoid or relieve DHD. Therefore, this program is expected to improve mental maladaptation and reduce suicidal ideation by having an effective on interventions for patients who feel hopelessness.

No statistically significant effects of the intervention on the POMS subscale scores were observed, possibly because patients had undergone psychological screening using the HADS scale prior to intervention. However, we consider relieving existential distress at an early stage to be important to prevent development to severe depression and anxiety in the future. Therefore, we find the Short-term EGP effective from a preventive point of view.

### Completion rate of the group

In the present study, 31 of 34 cancer patients completed intervention. We believe that this drop-out rate was low (8.8%), and the remaining participants of the Short-term EGP continued to meet irregularly after termination of intervention. This high completion rate suggested that subjects may have appreciated the benefits of the five-week group therapy intervention due to the support for existential distress relief and improvement of QOL in a relatively short period of time. Thus, Short-term EGP may benefit both patients and clinicians in labor, time, and medical expenses.

### Limitations and prospects of this research

Several limitations of the present study should be considered. First, the participants did not have severe depression. In the examination of the effects of the new Short-term EGP, to ensure safety this program was implemented with the aim of alleviating existential distress in patients who are not at the depression level. The results of the present study suggest that Short-term EGP is particularly effective for those patients who feel a degree of hopelessness, warranting further study on the effects of Short-term EGP on cancer patients who feel hopelessness with a higher degree of depression. Second, the present study did not enroll a control group. Future studies are required for comparing Short-term EGP with control groups consisting of patients who do not participate in such a group therapy.

## Conclusion

Short-term EGP intervention could be effective in helping patients relieve their existential distress. Some of the treatment effects were maintained a month after the end of the intervention. The program led to cognitive changes in daily life and life purposes. Such changes may help to improve the QOL. In addition, Short-term EGP could be particularly effective for those patients who feel hopelessness after cancer diagnosis. Therefore, this program is expected to improve mental maladaptation and reduce suicidal ideation by having an effective on interventions for patients who feel hopelessness.

## Data Availability

The datasets used and/or analyzed during the current study are available from the corresponding author on reasonable request.
